# House Servants

**Published:** 1869-01

**Authors:** 


### HOUSE SERVANTS.

A MAN who accumulated a large fortune during the war in handling national securities, erected for himself a costly mansion, and in connection with the number of servants necessary to the proper care of such a magnificent pile of buildings for the comfort of only himself and wife, the neighbors have called it "The ——— Boarding House."

There are in Fifth Avenue, and other less aristocratic localities, as well as in other large cities, many families of two or three persons, who maintain as many or more servants, who, having very little to do, become in time unfit for going into families who are unable to pay for servants who do not devote their whole time to the duties which they owe to their employers; hence a fruitful source of worry, irritation, and final ill health to many a faithful wife and daughter. But the rich have earned their money, and should be allowed to spend it in whatever way a true liberty may allow.

In all countries the tendency is for the rich to grow richer, and the poor poorer; especially is it so in large cities; and honored be they who, not being classed among the rich, have wisdom and moral courage enough to disregard mere appearances, and to conform their expenditures to what they can easily afford; to hire few, or even no servants, unless there is a pressing necessity for it.

The venerable Dr. Spofford, has made the following statement in reference to domestic health and its connection with the

PREMATURE BREAKING DOWN

of our wives and daughters; especially those living in villages or country places, where wages are much more moderate than in cities; and it merits the thoughtful consideration of a very large proportion of our population.

"The cost of a hired woman or girl in the house during the last seven years, has been on an average at least $1.50 a week, and her board $1.50 more, and the extra cost of the fuel and cooking, between its being used by a prudent wife or a servant who has no object or interest in saving, will make at least $50 more, making the extra expense of keeping a maid or doing one's own work, $200 a year, or in twenty years, which is a kind of average time for families to secure independence or break down, amounts to $4,000, to be made or lost by just this one item of domestic economy. But the writer and several of his neighbors have now kept house for fifty years, in which time the sum would amount to $10,000, a sum which would have swept off every vestige of property any of us could ever pretend to possess, and left us poor and destitute, or dead long since with disappointment, hardship and mortification."

KITCHEN AND PARLOR DAUGHTERS.

There is such a mixture of truth and error in the following, from the same source, that it is desirable to separate them.

"A great deal is said at the present day about education, and thousands of dollars of public money is expended to induce parents to spend their lives and their estates to keep their daughters in the parlor or at school, studying Latin, and French, and Algebra till they are eighteen or twenty years of age, when a great majority of them must become the wives of farmers and mechanics of moderate means, or remain forever single. No man but a millionaire, or great capitalist, can afford to marry a wife who has been educated to think herself a learned lady, to be always dressed for the parlor, and above the cares and labors of the kitchen; and every man of sense who expects to work with head or hands for a living, will select the young lady for a help-mate who can cook and make butter and cheese, and clothes, rather than the one who has a diploma, or who is remarkable for her proficiency in languages, mathematics and music. That kind or amount of education which creates a distaste for the labors and cares of a New England household, or a taste for dress above what fathers or husbands can afford, or which even absorbs all her own earnings, or which leads to a round of visiting in rich and genteel families, is in nine cases out of ten misplaced, and a source of endless chagrin and disappointment."

It is believed that a clear observation will prove, that a well-educated woman, even if brought up in wealth and luxury, is made by that very education, the more capable of adapting herself to a change of condition or circumstances ; her superior intelligence and her self-respect will but qualify her the better for learning and performing household duties. The very arrangment of the furniture of a house, the selection of its curtains and its carpetings, so that there shall be an appropriateness and a fitness and an agreement of the whole, requires an educated taste. No fool can ever be a good cook. All school education is to discipline the mind to habits of study, of close observation, of comparison and logical deduction, all ending in perfecting the judgment, and in enabling each one to think for himself, or for herself. Any study, whether of mathematics or language, ancient or modern, helps to form habits of thoughtfulness and sound judgment ; it is not the mere knowledge of these things which is sought after in the schools, but the habit of mental training which is acquired in their study.

### A GOOD COOK.

The essential qualifications of a good cook are attention and judgment, to watch an article of food while it is in process of cooking, and to be able to judge when it is properly cooked ; the word "properly," having reference to the nature of the food, whether it requires more or less cooking, to render it the better suited to the easy yielding up of its nutritious qualities to the system. In fact, ignorance and want of judgment are identical, are one and the same. Hence, to afford our daughters the greatest facilities for a thorough education, in all the branches of learning, is but the qualifying them for keeping house, for sweeping the floors, for making the beds, for using the sewing-machine, for cooking a dinner ; while the superior intelligence which the education has given, does but aid the mind to a dignified philosophical and Christian submission to changed circumstances, should such change occur; and if it does not, that same education will aid its possessor to grace the head of the table, to adorn the drawing-room and to elevate society.

It is within our own personal knowledge, and we could give the names and residences of ladies in New York city, who have never known poverty, and have had all the facilities for a thorough education which wealthy and intelligent parents could procure, who are model house-keepers, who exercise a cultivated taste in the furnishing of their dwellings; in the tidiness and appropriateness of their table appointments, and the easy management of their servants—in several cases we know of, not changing their help for years together—and at the same time, giving less wages than others; and why?

Because their intelligence gives them the ability to manage their servants; to attach them to them by holding them up to their duties on the one hand, and on the other, being considerate of their feelings and their welfare; giving them encouragement and praise when merited, and showing to them and requiring of them, the measure of respect belonging to the position of each.

The lessons of the article then are two ; one to the poor, the other to the rich. Let the poor hire as little help as possible; and let all remember, who are more favored in the social scale, that the better and more thorough the education of their daughters the more competent will they be to fill their sphere in after life whether in a cabin or a castle.

